# Subchondral insufficiency fracture of the knee: review of current concepts and radiological differential diagnoses

**DOI:** 10.1007/s11604-021-01224-3

**Published:** 2021-11-29

**Authors:** Junko Ochi, Taiki Nozaki, Akimoto Nimura, Takehiko Yamaguchi, Nobuto Kitamura

**Affiliations:** 1 Department of Diagnostic Radiology, Suita Tokushukai Hospital, 21-1, Senriokanishi, Suita-shi, Osaka 565-0814 Japan; 2 Department of Radiology, St. Luke’s International Hospital, 9-1, Akashi-cho, Chuo-ku, Tokyo, 104-8560 Japan; 3 Department of Functional Joint Anatomy, Graduate School of Medical and Dental Sciences, Tokyo Medical and Dental University, 1-5-45, Yushima, Bunkyo-ku, Tokyo, 113-8510 Japan; 4 Department of Pathology, Dokkyo Medical University Nikko Medical Center, 632 Takatoku, Nikko, Tochigi 321-2593 Japan; 5 Department of Orthopaedic Surgery, St Luke’s International Hospital, 9-1, Akashi-cho, Chuo-ku, Tokyo, 104-8560 Japan

**Keywords:** Subchondral insufficiency fracture, Spontaneous osteonecrosis of the knee, Subchondral plate, Meniscal root tear

## Abstract

Subchondral insufficiency fracture of the knee (SIFK) is a common cause of knee joint pain in older adults. SIFK is a type of stress fracture that occurs when repetitive and excessive stress is applied to the subchondral bone. If the fracture does not heal, the lesion develops into osteonecrosis and results in osteochondral collapse, requiring surgical management. Because of these clinical features, SIFK was initially termed “spontaneous osteonecrosis of the knee (SONK)” in the pre-MRI era. SONK is now categorized as an advanced SIFK lesion in the spectrum of this disease, and some authors believe the term “SONK” is a misnomer. MRI plays a significant role in the early diagnosis of SIFK. A subchondral T2 hypointense line of the affected condyle with extended bone marrow edema-like signal intensity are characteristic findings on MRI. The large lesion size and the presence of osteochondral collapse on imaging are associated with an increased risk of osteoarthritis. However, bone marrow edema-like signal intensity and osteochondral collapse alone are not specific to SIFK, and other osteochondral lesions, including avascular necrosis, osteochondral dissecans, and osteoarthritis should be considered. Chondral lesions and meniscal abnormalities, including posterior root tears, are also found in many patients with SIFK, and they are considered to be related to the development of SIFK. We review the clinical and imaging findings, including the anatomy and terminology history of SIFK, as well as its differential diagnoses. Radiologists should be familiar with these imaging features and clinical presentations for appropriate management.

## Introduction

Subchondral insufficiency fracture of the knee (SIFK) is a microfracture related to repetitive physiological stress on the knee joint. It is a common cause of knee pain in middle-aged and older people [1]. Insufficiency fractures are a type of stress fracture, and they tend to occur in the weight bearing joints of the lower leg, such as the femoral head and talar dome of the ankle joint, apart from the knee joint [2–4].

As SIFK is a type of fracture, patients often present with an acute onset of knee pain, which usually lasts for several months. If bone morphology is maintained without bone collapse, conservative management with non-weight bearing of the affected limb is often the first treatment choice. The lesion does not heal in some patients, and subchondral collapse associated with bone necrosis eventually occurs. Surgical management, such as joint replacement, is often required in advanced cases or affected individuals with osteoarthritis (OA) [5, 6]. Early diagnosis and appropriate management are important for improving the prognosis of SIFK. However, early-stage lesions often do not show any abnormalities on radiographs at the initial visit, leading to delays in proper treatment [7]. On the other hand, MRI is an excellent tool for the early detection of osteochondral abnormalities. Therefore, MRI should be considered for older people presenting with sudden onset knee pain and normal radiographs. With the increasing number of older people, there is an ever-increasing need for accurate assessment of their knee pain. Herein, we discuss the current concept of SIFK and its imaging findings, including the differential diagnoses that should be considered.

## History of SIFK

**Fig. 1:**
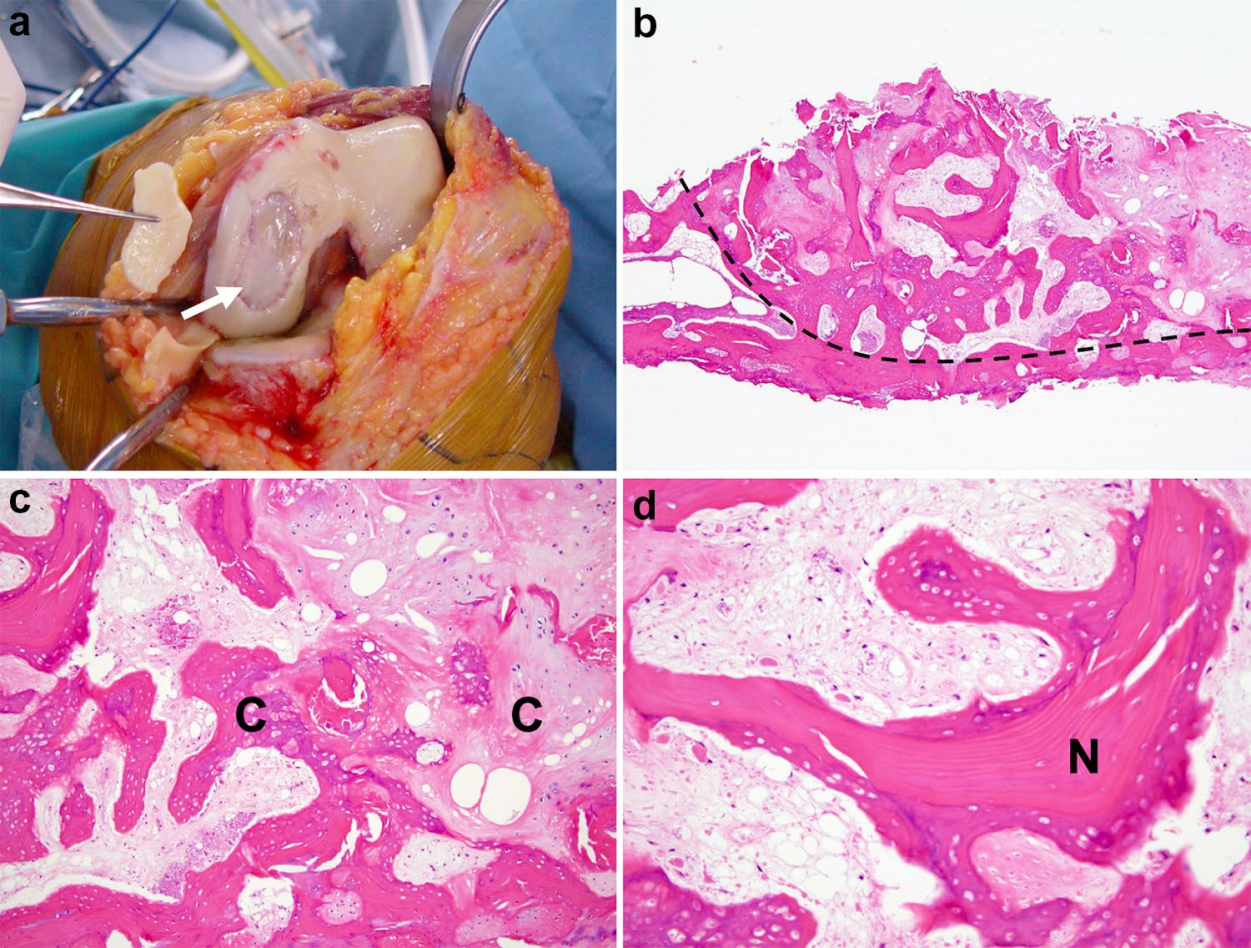
**a** Photograph obtained during total knee arthroplasty for SIFK in the medial femoral condyle. An osteochondral detachment from the subchondral bone can be seen (arrow). **b–d** Histology of subchondral insufficiency fracture (hematoxylin–eosin stain). **b** The lower surface indicates denuded subarticular bone of the distal femoral condyle, which is caused by secondary osteoarthritic change. The right field (right side of the dashed line) corresponds to an area of osteonecrosis (original magnification × 20). **c** The necrotic area shows marked callus formation (C), which indicates cellular appositional new bone and metaplastic cartilage formation (original magnification × 100). **d** Necrotic bone trabeculae (N) consisting of appositionally formed fiber bone on the original lamellar bone. The intertrabecular space is filled with loose fibrous connective tissue that is incompletely necrotic. These findings support a scenario in which a necrotic event occurs after preceding subchondral fracture (original magnification × 200)

SIFK was first described by Ahlbäck et al. in 1968 as “spontaneous osteonecrosis of the knee (SONK).” They concluded that it was a primary osteonecrosis because osteonecrosis was observed on the specimen, although no apparent cause could be identified [8]. In the 1990s, the concept of subchondral insufficiency fracture (SIF) of the femoral head gradually spread. The pathogenesis of SONK was also reconsidered, and in 2000, Yamamoto et al. reported in their histological study that the true nature of SONK is a subchondral fracture, while osteonecrosis is a secondary finding [9]. Similar reports followed their conclusions [6, 10, 11], and it is now widely accepted that SONK is the end-stage of the subchondral fracture and is a part of the spectrum of SIFK (Fig. 1). Considering the history of the terminology, both SONK and SIFK have been used to describe this disease. Recently, some researchers have suggested that the word “spontaneous” in SONK does not reflect the pathogenesis and that the term “SONK” should no longer be used [1, 12]. Although the pathological mechanism leading from fracture to bone necrosis has not been elucidated, it has been suggested that increased intraosseous pressure and diminished local blood circulation associated with subchondral microfractures may be involved in osteonecrosis [13].

## Anatomy

**Fig. 2:**
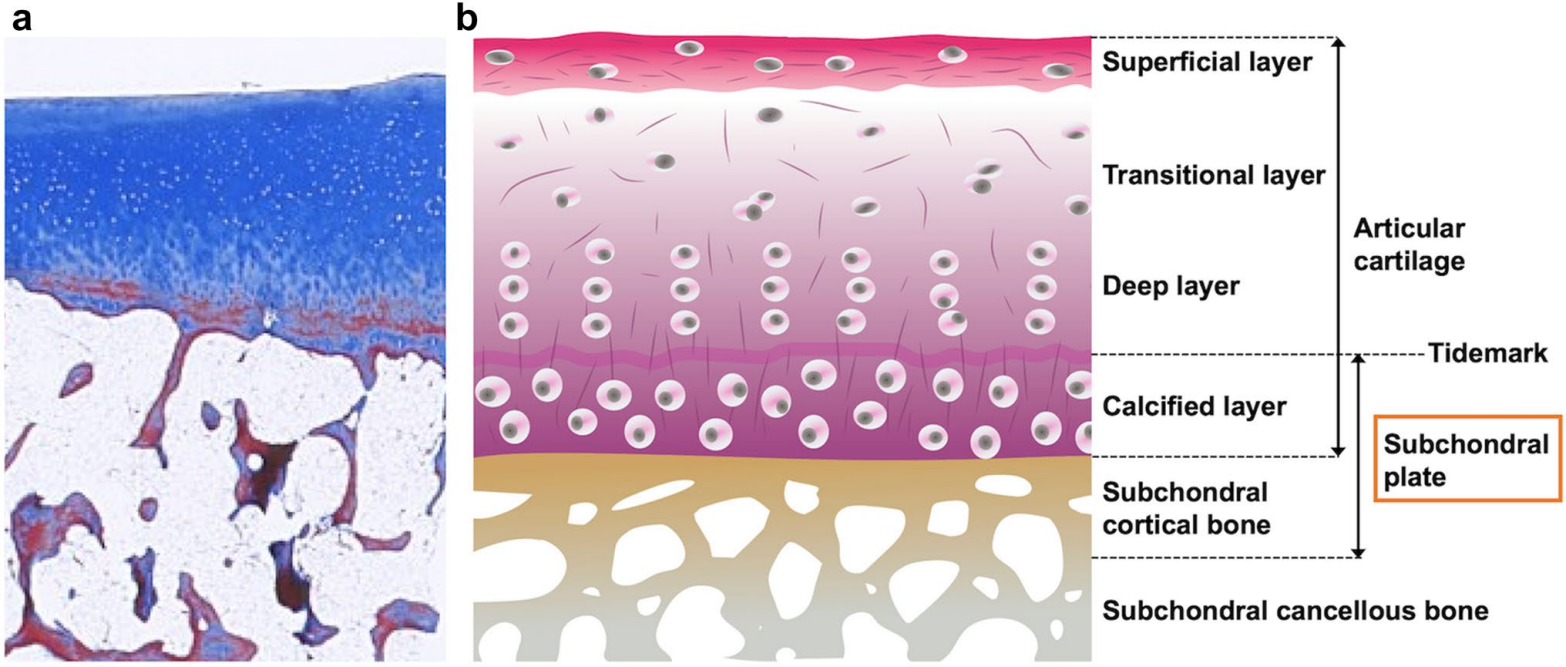
**a** Frontal section of the tibial plateau (Masson trichrome stain). **b** Detailed schematic drawing of the articular cartilage and underlying subchondral bone

The target lesion of SIFK is the epiphyseal lesion of the knee joint. The epiphysis consists of articular cartilage, subchondral cortical bone, and underlying subarticular trabecular bone. Articular cartilage comprises an extracellular matrix consisting of a large amount of water, collagen, proteoglycans, and a small number of chondrocytes [14]. Articular cartilage in adults consists of four layers with different cellular profiles and collagen organization as follows: the superficial, transitional, deep, and calcified layers, in order from the articular surface. Between the deep and calcified layers of articular cartilage, an area known as the tidemark exists, which stains darkly with hematoxylin [14, 15]. The tidemark corresponds to the mineralization front and has several biomechanical functions. The deepest calcified layer and the underlying subchondral cortical bone are collectively referred to as the subchondral plate, which supports the superficial cartilage layers (Fig. 2) [16]. The subchondral plate is not uniform in thickness, and it is thicker at the weight bearing area and thinner at the peripheral region of the condyle. It is difficult to distinguish between the two layers of the subchondral plate in MRI used in daily practice, and a single linear low-signal band can only be recognized on T2-weighted image (T2WI) or proton density-weighted image (PDWI) [17]. In the field of radiology, the term “subchondral bone” or “subchondral region” is often used ambiguously and generally refers to the subchondral plate and nearby cancellous bone [13]. Diseases of the articular cartilage and subchondral bone are collectively referred to as osteochondral lesions.

## Clinical presentation

SIFK is a type of insufficiency fracture that occurs with the sudden onset of knee pain without any history of trauma. Patients can often precisely recall when and what they were doing when the pain started. The pain increases with weight bearing and exercise of the knee and often persists even at rest, including at night. Physical examination reveals tenderness in the affected area, and joint effusion is commonly observed [7, 8, 18–20]. These symptoms are sometimes similar to other medical conditions, including meniscus and cartilage injuries [17, 21, 22].

SIFK is more common in women over 50 years of age [19, 21, 23, 24]. Plett et al. reported that 64.4% (47/73) of 73 SIFK patients were women [5]. Advanced lesions have also been reported to be more common in women [24]. Low bone mineral density (BMD) associated with menopause may play a role in why SIFK is more common in older women.

SIFK is most common in the weight bearing area of the medial femoral condyle [25]. It also infrequently occurs in the medial tibial condyle, lateral femoral condyle, and lateral tibial condyle compared with the medial femoral condyle [26–28]. Wilmot et al. analyzed 74 cases of SIFK and found that 64.9% (48/74) of the cases were in the medial femoral condyle, 16.2% (12/74) each in the lateral femoral and medial tibial condyles, and 2.7% (2/74) in the lateral tibial condyle, with a preference for the central one-third of each condyle (70–77%) [24]. Most cases are unilateral, but multiple or bilateral condyles may be affected simultaneously or at different times [19, 29]. Reddy and Frederick reported in a cadaveric study that the medial femoral condyle had a poorer intraosseous blood supply with watershed areas compared with the lateral femoral condyle, which may be related to the preference for the medial femoral condyle [30]. It may also be related to differences in bone strength at each condyle [31] or the frequency of meniscus injuries, which will be discussed later.

## Imaging findings

### Radiography

**Fig. 3:**
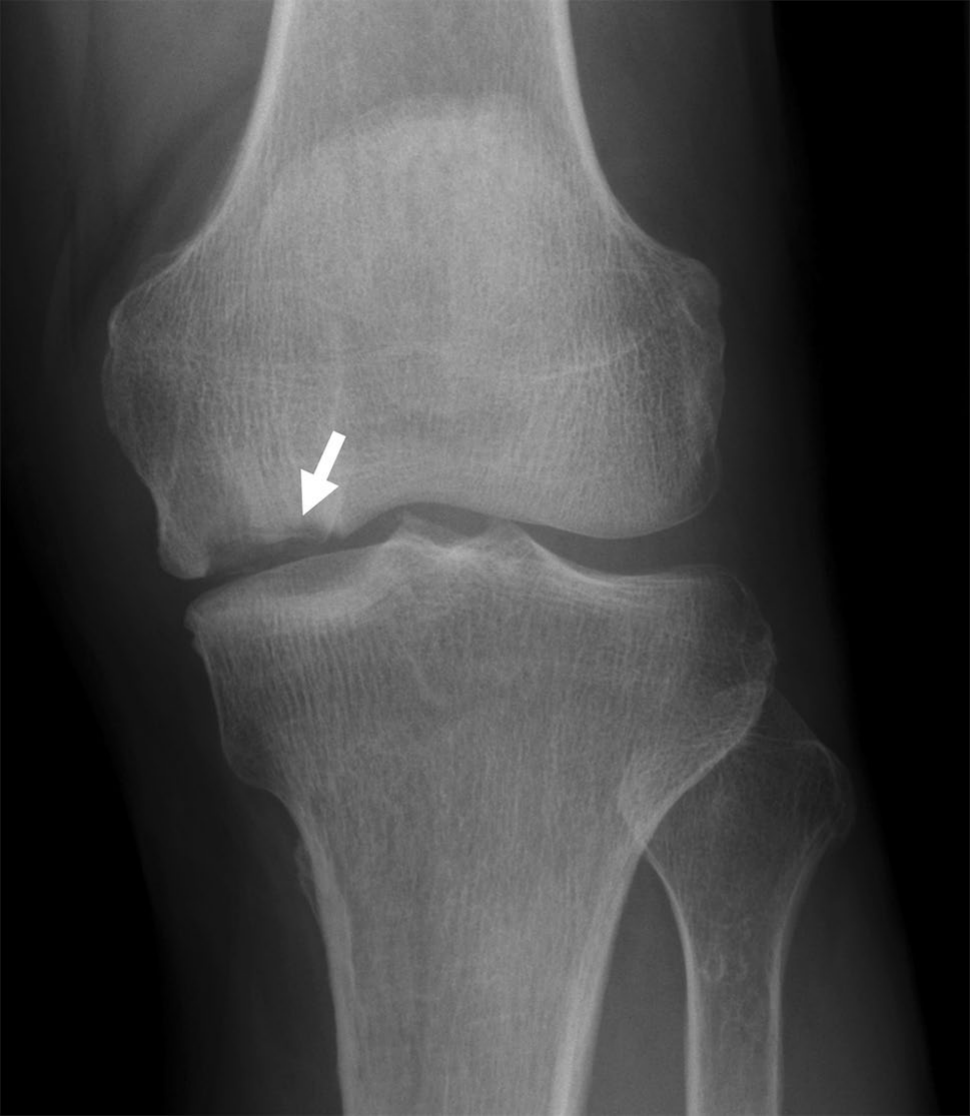
Plain radiograph of SIFK in a 75-year-old female patient. Saucerized defect (arrow) of the epiphysis can be observed in the medial femoral condyle of the left knee

Radiography of the lesion is usually performed to evaluate bony morphology, determine the most appropriate therapeutic strategy, and differentiate SIFK from other diseases. The fracture line in SIFK is often not clear on radiographs; as a result, their initial imaging evaluations are often negative. As the lesion progresses, radiographic abnormalities, including osteochondral defects or deformities of the epiphyses, are visualized (Fig. 3) [7, 8, 23, 32]. In 1979, Koshino et al. advocated a clinical classification system for SONK based on symptoms and radiographic findings. Since SONK is now considered part of the SIFK spectrum as described above, it is reasonable to apply the Koshino classification to SIFK. The classification is as follows: stage 1, patients with knee symptoms without any radiographic findings; stage 2, patients with bone flattening and subchondral radiolucencies; stage 3, patients with subchondral collapse; and stage 4, patients with degenerative osteoarthritic changes (Table 1) [33]. The Koshino classification is still often used as a reference for determining treatment strategies.

**Table 1:** Koshino classification of SIFK

Stage	Radiographic finding
Stage I	Normal radiograph
Stage II	Radiolucency in subchondral weight-bearing area
Stage III	Expanded lucent area surrounded by sclerosis, subchondral bone collapse
Stage IV	Osteophytes and osteosclerosis on affected condyle

### Magnetic resonance imaging

**Fig. 4:**
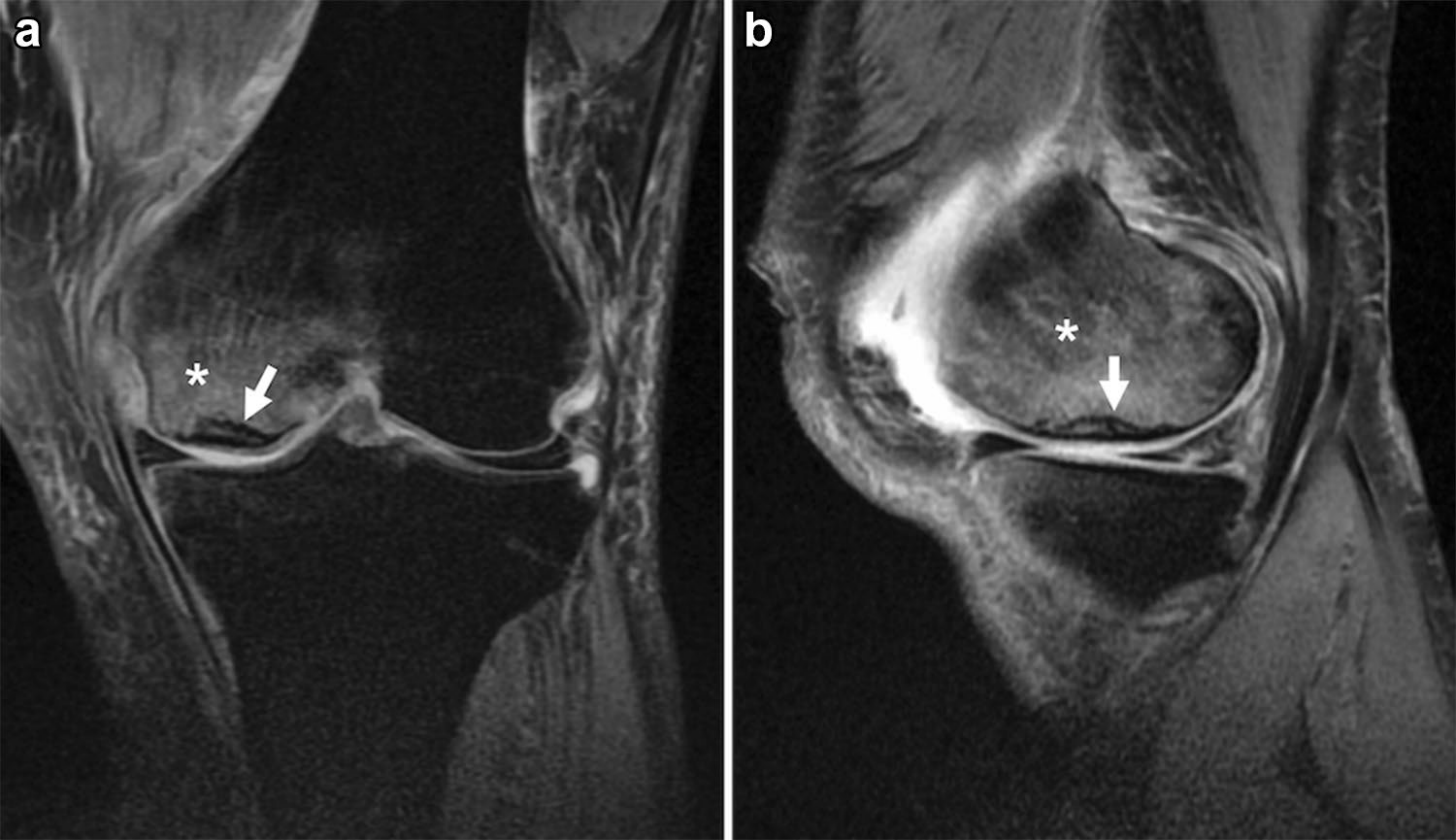
MRI of a 50-year-old man with a complaint of sudden left knee pain. **a** Coronal fat-suppressed proton density-weighted image (FS-PDWI), and **b** sagittal FS-PDWI show extensive bone marrow edema-like signal intensity over the medial femoral condyle (asterisk). A subchondral hypointense line is observed a few millimeters above the subchondral plate (arrow). Note that the line is almost parallel to the subchondral plate and open-ended in the medial part

Radiographs are not sensitive for the early diagnosis of SIFK, while MRI is excellent for detecting osteochondral lesions and is optimal for early diagnosis when SIFK is suspected [32, 34]. The characteristic findings on MRI are a bone marrow edema-like signal intensity and a subchondral hypointense line of the affected condyle (Fig. 4) [9, 35, 36].

The term “bone marrow edema” was first used by Wilson et al. in 1988 to describe abnormal bone marrow signal intensity on MRI. They found subtle low signal intensity on T1-weighted image (T1WI) and subtle high signal intensity on fat-suppressed T2WI in patients complaining of knee pain. They believed that it represented bone marrow edema, but there was no histological evidence [37]. With the spread of MRI, this finding has been observed in knees with various conditions, including SIFK, OA, trauma, inflammation, and tumors [38]. Pathologically, this finding has been proven not to be mainly edema, but a mixture of lymphoid infiltrates, fibrosis, increased vascularization, blood products, granulomatous foci of fragmented bone, and cartilage debris in various proportions [11, 12, 39–43]. For this reason, it is increasingly being referred to as bone marrow lesions, bone marrow edema-like lesions, or bone marrow edema-like signal intensity instead of “bone marrow edema” [12]. As mentioned above, this bone marrow edema-like signal intensity is seen in various conditions and is not specific to SIFK. However, it is characteristic of SIFK in that it extends over a wide area of the affected condyle [17, 35]. Ramnath et al. reported that 82% (9/11) of SIFK patients had this signal change, extending over two-thirds of the condyle [36]. Although there is no pathological proof in the literature that explains the reason for this widespread signal abnormality in SIFK patients, it may be due to the reactive changes in bone related to fracture as well as mechanical stress on bone associated with osteochondral and meniscal injuries in SIFK. Nevertheless, the extent of this bone marrow edema-like signal intensity on initial MRI is not associated with the prognosis of SIFK [5, 35].

**Fig. 5:**
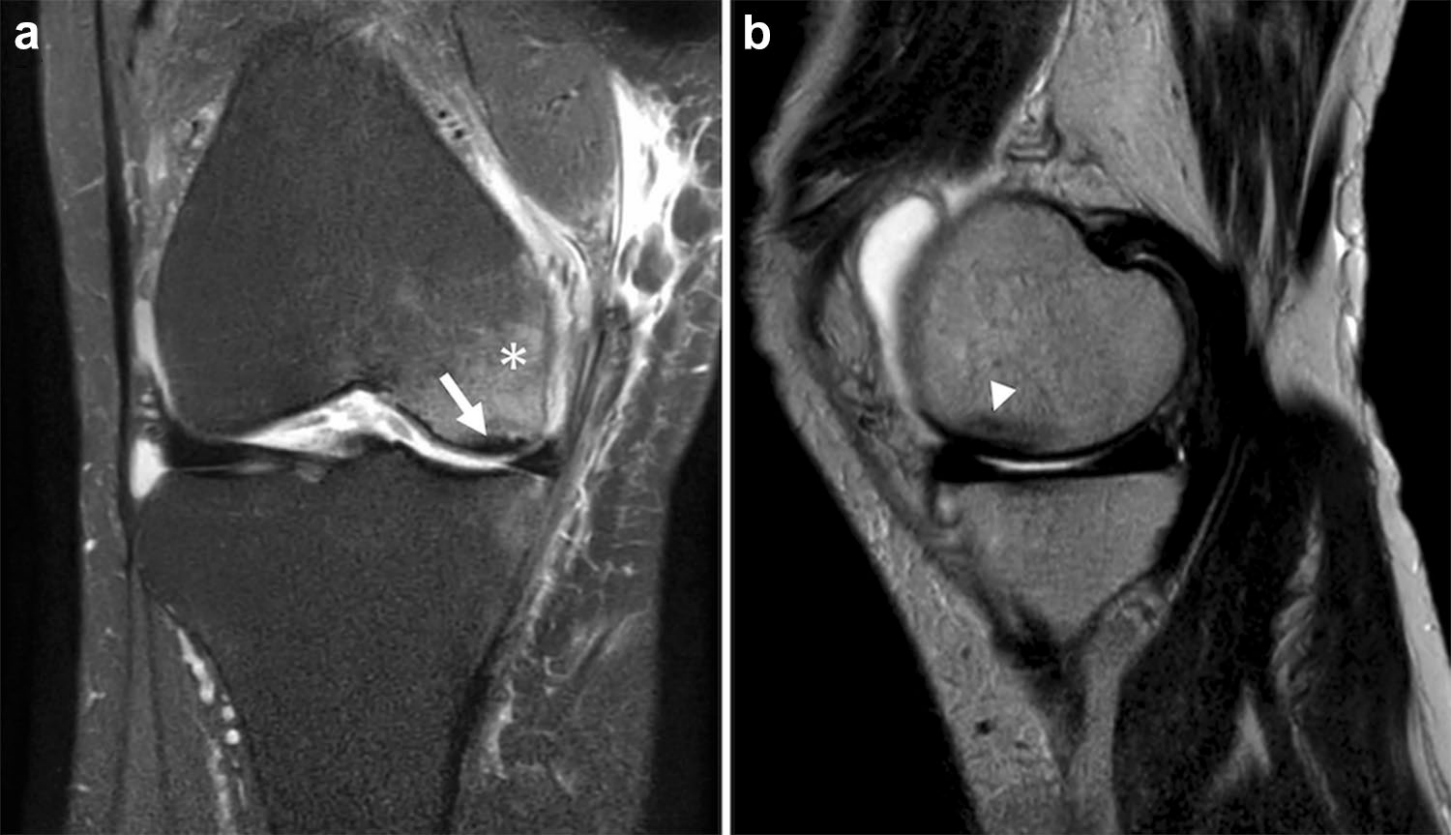
MRI of a 73-year-old female patient with SIFK in the medial femoral condyle of the right knee. **a** Coronal FS-PDWI shows extensive bone marrow edema-like signal intensity over the medial femoral condyle (asterisk) and a subchondral area of low signal intensity (arrow), which is integrated with the subchondral plate and resembles a thickened subchondral plate. Note the medial meniscal extrusion which is an associated finding. **b** A sagittal T2-weighted image (T2WI) showing a subtle hypointense line (arrowhead) above the subchondral area of low signal intensity

The subchondral hypointense line on T2WI and PDWI is a more characteristic finding of SIFK. Unlike acute trauma, the fracture line in SIFK usually shows a low signal in any sequence, including fluid-sensitive MR sequences [12]. Histologically, this hypointense line corresponds to a fracture callus, thickened collapsed bone trabeculae, reactive cartilage, and granulation tissue [6, 9, 44]. This subchondral hypointense line often runs roughly parallel or curvilinear to the subchondral plate a few millimeters away from the epiphyseal surface. It may also be discontinuous or open-ended [17, 45]. Similarly, a subchondral area of low signal intensity may be observed, which is a mixture of fracture callus, granulation tissue, and secondary osteonecrosis of the superficial layer. This subchondral area of low signal intensity is often integrated with the subchondral plate, and radiologists should not overlook it because it resembles a thickened subchondral plate (Fig. 5) [17].

**Fig. 6:**
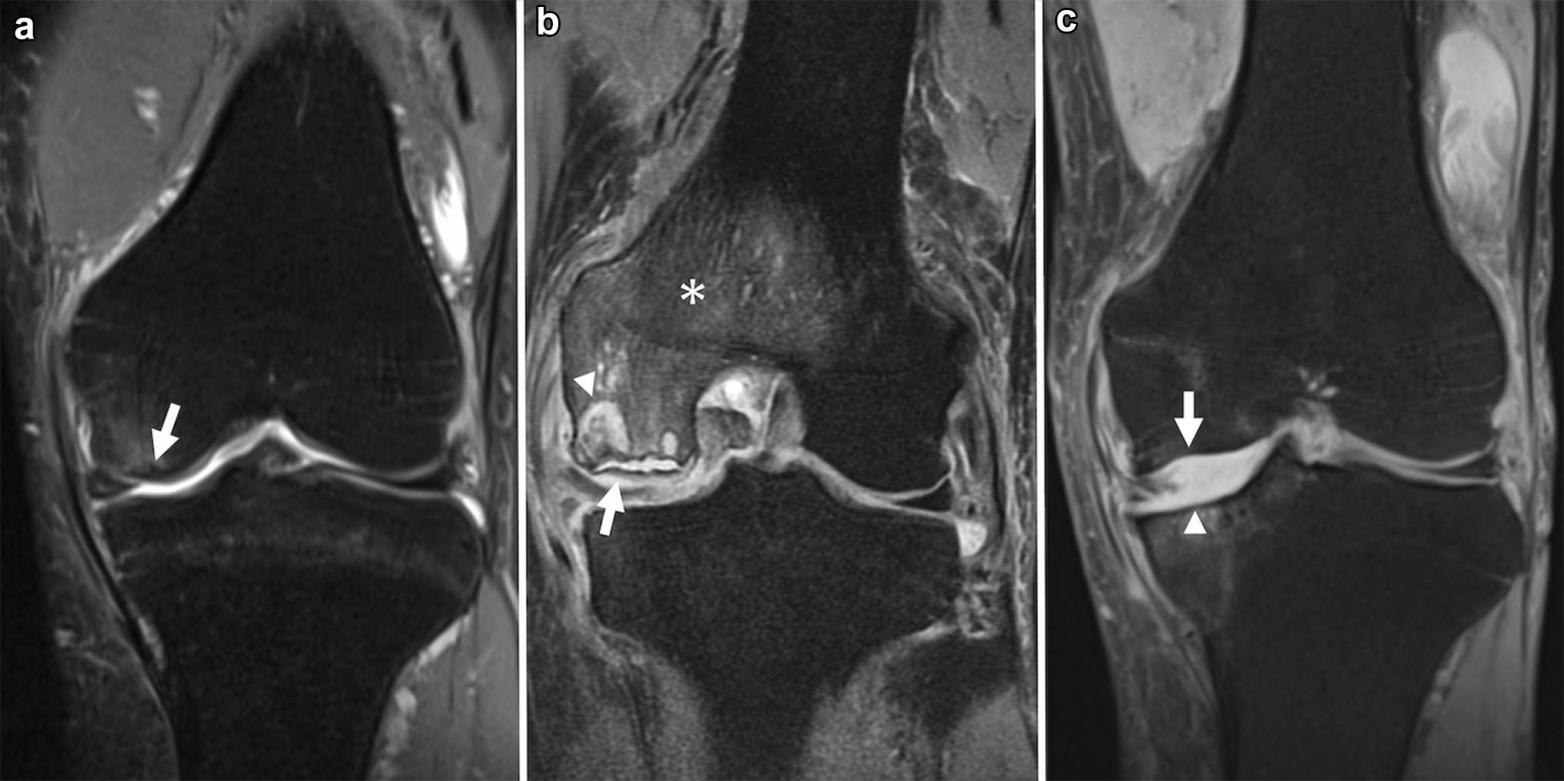
Coronal FS-PDWI of a 71-year-old female patient with SIFK in the medial femoral condyle of the left knee. **a** The initial MR image shows a subchondral hypointense line (arrow) with subtle bone marrow edema-like signal intensity around the lesion. **b** An MRI obtained 6 months later shows a fluid-filled cleft under the subchondral plate (arrow) and a large cyst with necrotic bone fragments beneath the lesion (arrowhead). The bone marrow edema-like signal intensity appears to expand over the medial femoral condyle (asterisk). **c** An MRI obtained 4 years later shows an osteochondral defect of the condyle (arrow). There is also an osteochondral defect on the tibial side (arrowhead) because the patient developed another SIFK in the medial tibial condyle during the disease

In addition, a slight contour deformity or flattening of the epiphysis could be visualized, which reflects a fracture of the subchondral plate [17]. In advanced cases, a fluid-filled cleft under the subchondral plate or an apparent osteochondral defect of the bone may be seen, corresponding to bone collapse or dissection associated with osteonecrosis after the fracture (Fig. 6) [46].

### Bone scintigraphy

Bone scintigraphy shows uptake at the lesion site, but its sensitivity and specificity are low [7, 47]. Therefore, bone scintigraphy is no longer recommended for the diagnosis of SIFK in the current MRI era.

## Associated findings

The etiology of SIFK is considered multifactorial, and several risk factors or associated findings have been reported to date.

### Bone mineral density

SIFK is a type of insufficiency fracture. Based on this definition, low BMD can be considered a risk factor for SIFK. Several studies have reported an association between low BMD and SIFK [5, 19, 48, 49]. However, Nelson et al. found that only 16% (5/32) of SIFK patients were classified as osteoporotic. In comparison, 44% (14/32) of patients showed normal BMD, suggesting that low BMD is not the main risk factor for SIFK [50]. To date, the relationship between BMD and SIFK is still controversial, and there is no clear evidence regarding a threshold BMD that is associated with the risk of SIFK.

### Meniscal abnormalities

**Fig. 7:**
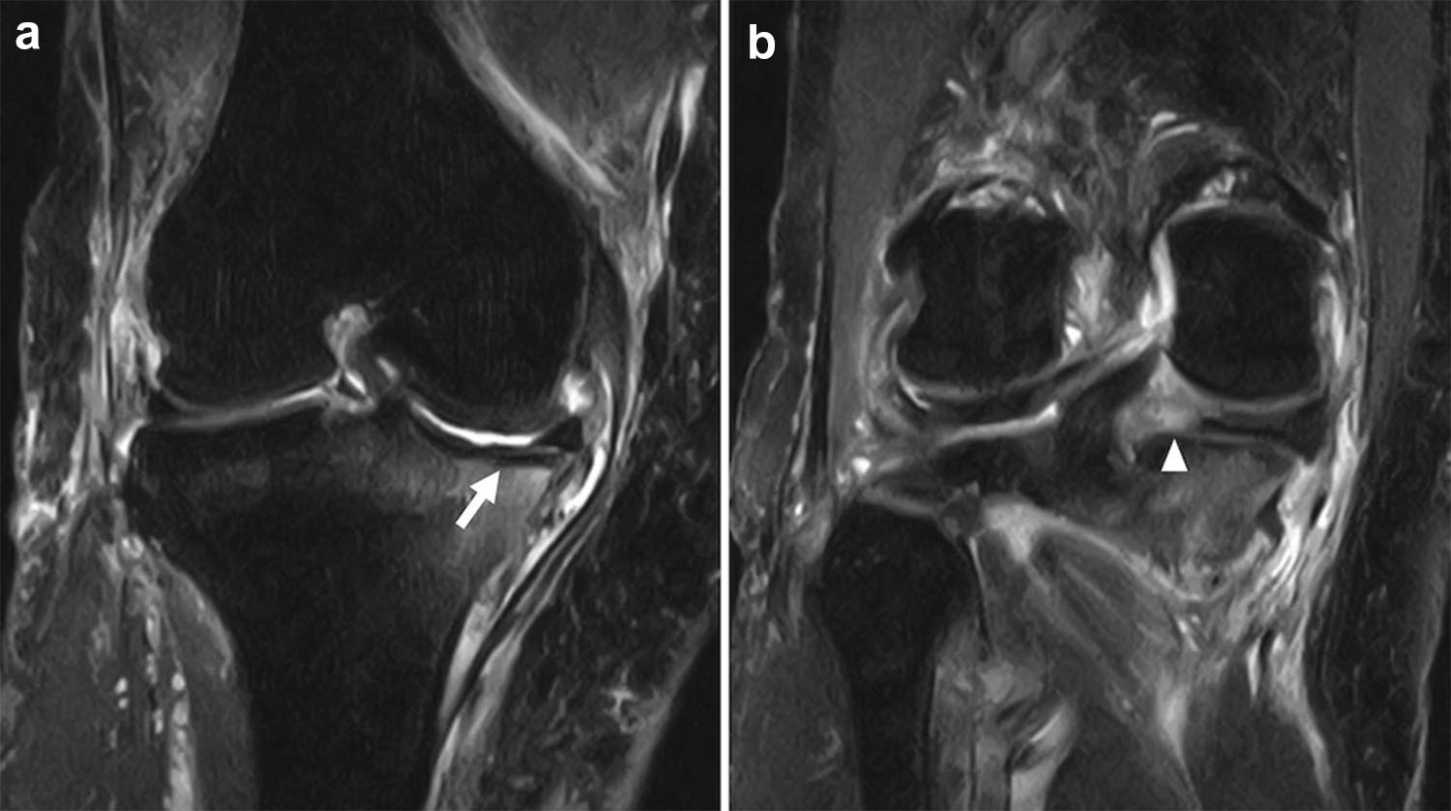
Coronal and sagittal FS-PDWI of a 79-year-old female patient who complained of a sudden worsening of knee pain during follow-up for osteoarthritis of the right knee. **a** A subchondral hypointense line (arrow) with bone marrow edema-like signal intensity can be seen in the medial tibial condyle. **b** Note the posterior root tear of the medial meniscus (arrowhead)

Many retrospective studies have mentioned the association between meniscal tears and SIFK, and meniscal abnormalities likely play a role in the development of the disease [1]. Most of these meniscal abnormalities are medial meniscal tears, often ipsilateral to the SIFK [5, 28, 36, 51, 52]. The most common tear is a posterior root tear, followed by a radial tear in the posterior horn (Fig. 7) [6, 28, 53, 54]. There are some reports of an association between SIFK and medial meniscal extrusion (Fig. 5) [6, 53–55], as well as some reports about SIFK and meniscectomy [11, 56, 57].

The meniscus plays an essential role in the movement and stability of the knee joint [58, 59]. The meniscus is a C-shaped structure consisting of fibrocartilage, which is firmly attached to the tibia at the anterior and posterior roots. In the deep central main layer of the meniscus, collagen fibers run circumferentially and convert the vertical load on the knee joint into circumferential hoop stress [60]. Owing to this structure, called “the hoop mechanism,” the load applied to the knee is distributed in the circumferential direction of the meniscus. In particular, the posterior root of the meniscus is known to contribute more to meniscal stability than the anterior root. Tears in the attachment of the posterior root of the meniscus are considered a risk factor for meniscal extrusion [53, 61, 62]. If this hoop mechanism is disrupted due to meniscal tears, extrusion, or resections, the contact pressure on the loading surface of the knee increases, which may lead to the development of SIFK [63].

### Osteoarthritis

Several reports have shown an association between knee OA and SIFK [5, 10, 64]. Recently, Allam et al. reported that the degree of chondrosis was related to the severity of SIFK [64]. As meniscus has a role to protect the knee articular surface from mechanical stress, meniscal injury is also known to be related to the development of knee OA [63]. Plett et al. reported that 75.7% (53/70) of SIFK patients had overlying full-thickness cartilage loss, and 94.1% (64/68) had ipsilateral meniscal injuries [5], and meniscal injury may be associated with the development of both SIFK and OA.

### Overweight/obesity

Studies on the relationship between patient weight and SIFK have shown that overweight patients have more stress on the joints in the lower limb. Zanetti et al. found that 40.6% (13/32) of SIFK patients were overweight or obese (body mass index > 25.0 kg/m^2^) [48]. However, it has been recently reported that body weight is not associated with the prognosis of SIFK [46].

## Prognosis and treatment

### Prognostic factor

The prognosis of patients with SIFK varies. Some patients recover with conservative management, while others fail to heal, resulting in necrosis of the subchondral bone of the lesion and eventual need for knee joint replacement surgery [5, 17, 46]. In hip SIF, the prognosis is influenced by factors including age, weight, BMD, fracture size, patient activity, and initial treatment [3]. These factors may also be associated with the prognosis of SIFK.

In SIFK, lesion size has been reported to be related to prognosis in several publications. Generally, the larger the lesion, the worse the prognosis, although there are several methods for measuring lesion size, and there is no standardized method of measurement or cutoff value for determining a poor prognosis [21, 64]. Aglietti et al. measured the maximum diameter of the lesion in two directions, anteroposterior and transverse, on plain radiographs and reported that the prognosis was poor if the lesion area was more than 5 cm^2^ or if the maximum width ratio of the lesion in the anteroposterior view exceeded 40% of the affected condyle [19]. Similarly, Lotke et al. found the cutoff ratio of the width of the lesion to exceed 50% of the condyle in those with a poor prognosis [20]. Lecouvet et al. reported in their study using MRI that subchondral areas of low signal intensity with a length of more than 14 mm or a thickness of more than 4 mm on T2WI were associated with a poor prognosis [35].

Osteochondral defects in SIFK are considered irreversible, and the prognosis of patients with this finding is unfavorable [35]. Recently, Sayyid et al. proposed an MRI grading system for SIFK to evaluate its prognosis [46]. They defined low-grade lesions as those with only bone marrow edema-like signal intensity and fracture lines on initial MRI, and high-grade lesions as those with cystic changes in the subchondral bone or osteochondral defects. They mentioned that high-grade lesions were more frequently associated with posterior meniscal root tears, moderate or severe meniscal extrusion (> 3 mm), and severe chondrosis. In 92.7% (38/41) of patients with low-grade lesions, more than 50% improvement in the range of bone marrow edema-like signal intensity in the follow-up MRI was noted within 1 year, suggesting that changes in bone marrow edema-like signal intensity over time may also reflect the prognosis of SIFK (Fig. 6).

### Treatment

Conservative management is the first choice in the early stages of SIFK. It includes protected weight bearing, insole therapy, administration of non-steroidal anti-inflammatory drugs, and, in some cases, bisphosphonates. Bisphosphonates are known to act on osteoclasts to inhibit bone resorption and are widely used for osteoporosis. Although bisphosphonates were shown to be effective in an animal study, Meier et al. found no efficacy in a randomized, placebo-controlled trial in humans, and their effectiveness for SIFK is controversial [65]. Recently, teriparatide, a drug for osteoporosis, has attracted attention regarding SIF treatment. Teriparatide is a recombinant parathyroid hormone that activates osteoblasts and promotes bone formation [66–69]. Teriparatide has been reported to be more effective than bisphosphonates in osteonecrosis of the femoral head [70] and has also been reported to be effective in SIFK [71, 72]. It usually takes several months for SIFK to heal with conservative management. Yates et al. followed 20 conservatively treated SIFK patients and reported that it took an average of 4.8 (range 3–8) months for symptoms to disappear and an average of 8 (range 3–18) months for the MRI to normalize [72].

When conservative management fails to improve, surgical treatment is selected according to the size and progression of the lesion [73]. Both MRI and radiographic evaluations are important in the treatment decision-making process. The Koshino classification system for radiographs is often used since it was the first method developed to classify diseases according to the degree of progression and determine the most appropriate treatment. MRI is often used to determine the size of the lesion, differentiate between other types of osteochondral lesions, and evaluate concomitant meniscal or chondral abnormalities. For patients with relatively preserved bone morphology under stage 2 of the Koshino classification, joint-preserving surgeries such as bone grafting, osteochondral grafting, core decompression, and high tibial osteotomy are indicated [21, 33, 74]. Lesions classified as stage 3 or higher are considered to have little chance of cure. Unicompartmental knee arthroplasty is a good indication if the lesion is confined to a single condyle. High tibial osteotomy may also be indicated if the patient is young and the lesion is limited to the medial side or if the patient has medial OA along with SIFK without osteochondral collapse [75, 76]. Total knee arthroplasty is the treatment of choice when multiple condyles are affected or when patients develop severe knee OA along with SIFK [73, 74].

## Differential diagnosis

Transient bone marrow edema syndrome (transient BMES) of the knee is a rare, self-limiting disease that can be difficult to differentiate from SIFK. Other osteochondral lesions include avascular necrosis (AVN), osteochondral dissecans (OCD), knee OA, and acute trauma, such as tibial plateau fractures. Since these osteochondral lesions often present with similar imaging findings, it is important to know the clinical conditions and radiological features to differentiate these diseases.

### Transient bone marrow edema syndrome

Transient BMES is a rare but reversible condition characterized by pain and bone marrow edema-like signal intensity on MRI. Various terms have been used to describe this condition, including transient osteoporosis or regional migratory osteoporosis when it migrates to a different joint or within the same joint over several months [77–79]. Although transient BMES is much rarer than SIFK, it may be overlooked because of its self-limited nature. Transient BMES affects the weight-bearing joints of middle-aged men and young women exclusively in the third trimester of pregnancy or postpartum period [80].

**Fig. 8:**
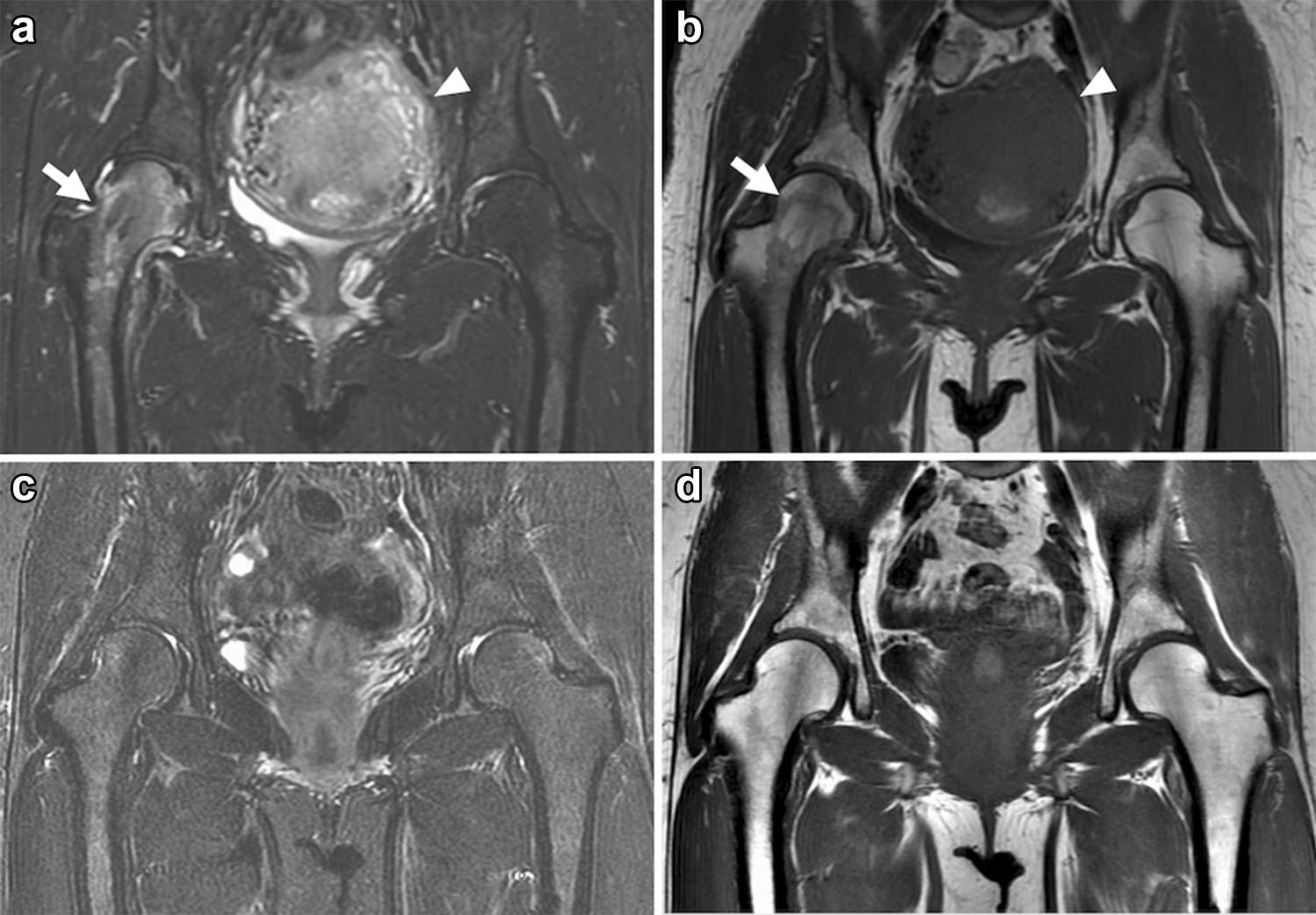
Coronal FS-PDWI and T1WI of a 40-year-old female patient in the postpartum period with transient BMES in the right hip. MRI was performed on the 12th day post-cesarean section for the complaint of severe right hip pain. **a**, **b** Initial MRI shows a diffuse bone marrow edema-like lesion extending from the right femoral head to the trochanteric region (arrow). No other obvious abnormal findings are noted, and the postpartum uterus is still enlarged (arrowhead). **c**, **d** The patient was treated conservatively, and her symptoms improved. A follow-up MRI taken 4 months later shows the disappearance of the bone marrow edema-like lesion in the right hip

Transient BMES is primarily characterized by extensive bone marrow edema-like signal intensity on MRI, and focal osteopenia may be present on radiographs in 3–6 weeks after the onset of symptoms. Most cases occur in the hip, and there are only a few reports of imaging findings in the knee [81]. Usually, diffuse bone marrow edema-like signal intensity is the only finding on MRI, and most cases are not associated with meniscal tears or other morphologic alterations (Fig. 8) [78, 82]. Conversely, some authors have reported that more than 77% of patients with transient BMES in the hip had subchondral line or subchondral focal hypointensity on the initial MRI [83, 84], which suggested that transient BMES may be related to a previous subchondral fracture healed with conservative treatment. However, many factors remain unclear, such as the pathogenesis of osteopenia, mechanism of migration in some cases, and the reasons healthy, young to middle-aged patients are more affected. Considering the findings of the previous studies, it may be difficult to clearly distinguish transient BMES from SIFK based on images alone. Both the patient background and clinical course should be considered to obtain a proper clinical diagnosis.

### Avascular necrosis (bone infarction)

AVN is referred to as bone infarction of the epiphyses caused by ischemia [17]. It is an entirely different condition from SIFK/SONK, but some previous studies confused SIFK/SONK with AVN. Although AVN is more common in patients in their 30s and 40s, the presence of risk factors plays a more significant role in its occurrence than age [45, 73]. There are several risk factors for AVN, including alcohol consumption, steroid therapy, tobacco abuse, connective tissue disease, caisson disease, sickle cell anemia, and Gaucher’s disease. Vaso-occlusive effects, high intraosseous pressure, or altered fat metabolism due to these factors are thought to involve bone ischemia [85–88]. Unlike AVN of the hip joint, AVN of the knee joint rarely develops after a fracture. The onset of AVN is typically slower than that of SIFK. The pain of infarction is relatively non-severe, and some cases are asymptomatic [17, 89].

**Fig. 9:**
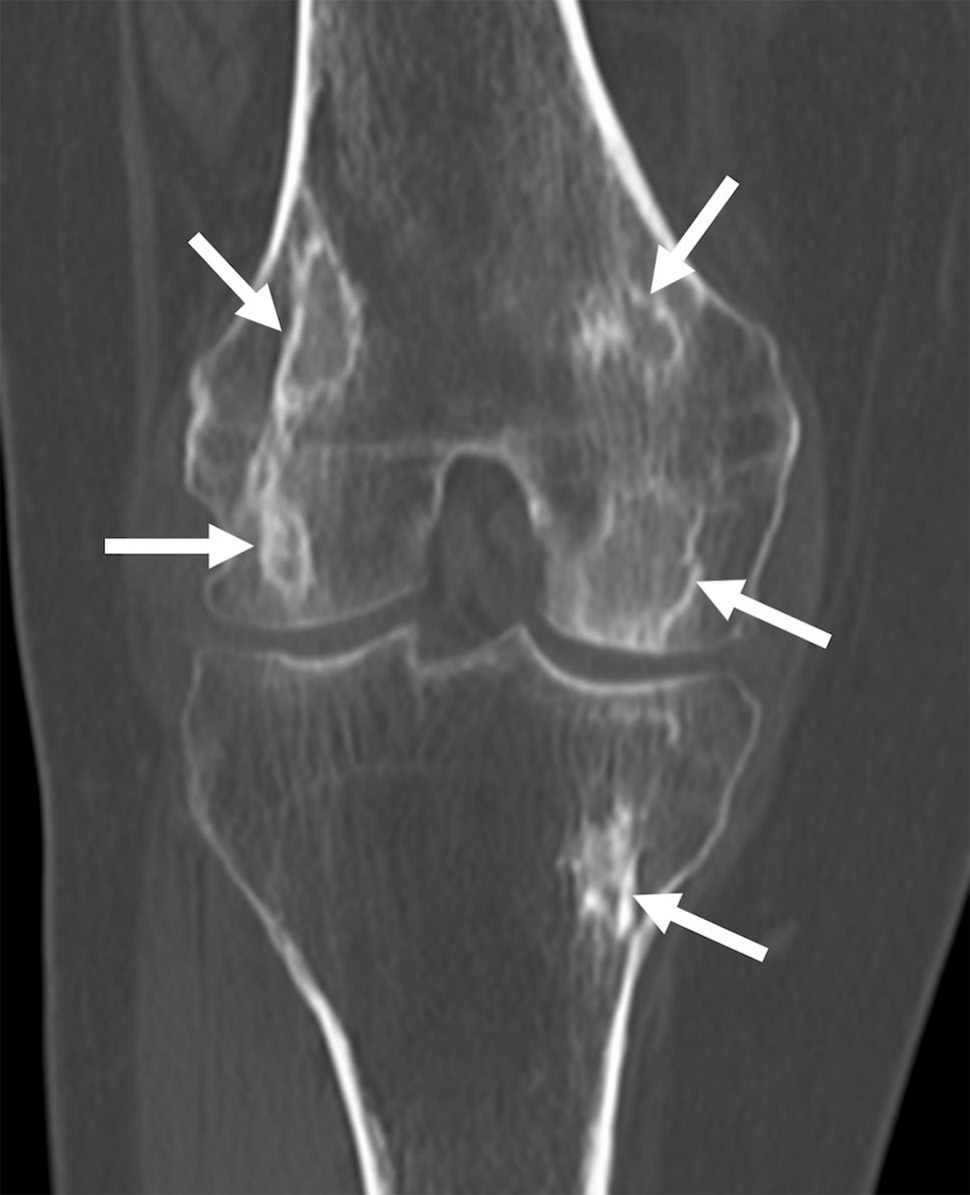
A 60-year-old female patient with sarcoidosis who had been treated with steroids for years. A reformatted CT coronal image shows multiple lesions with a serpiginous sclerotic border (arrows), which corresponds to lesions of AVN/bone infarction

**Fig. 10:**
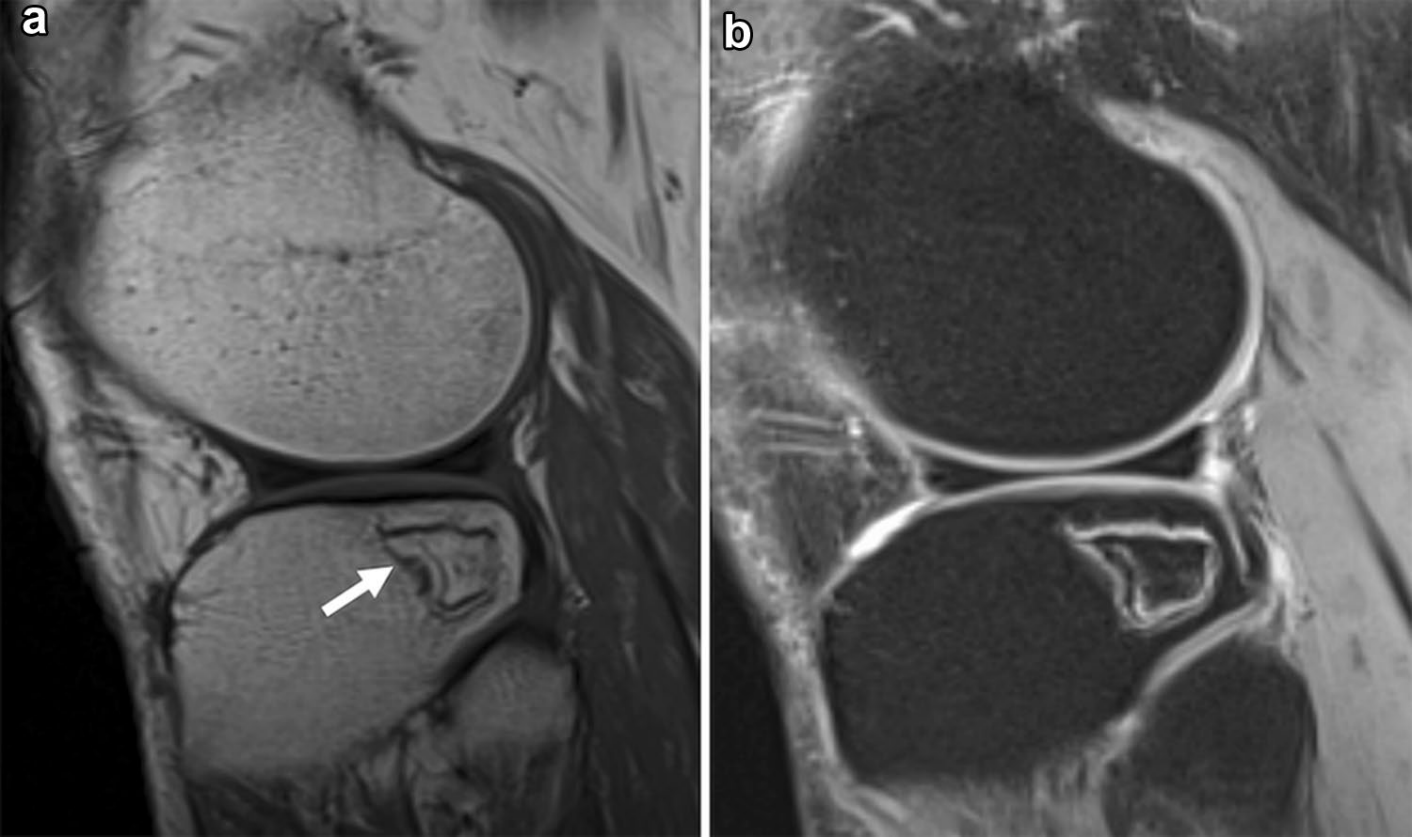
A 60-year-old male patient with AVN. **a** A sagittal T2WI shows necrotic areas surrounded by a high and low-signal-intensity rim (arrow). The rim is displayed as a “double-line sign”. **b** A sagittal FS-T2WI shows that the signal of the center of the lesion is preserved with a fatty marrow signal, although it is composed of necrotic bone marrow tissue

AVN appears as a sclerotic geographic change on plain radiography and CT (Fig. 9). Sclerotic changes surround the necrotic area and are often serpentine in shape. On MRI, the irregularly shaped necrotic area is circumscribed with a distinct rim. This rim often consists of a double line of low and high signals on T2WI called a “double-line sign” and is regarded as a characteristic finding of AVN (Fig. 10) [17, 88]. This sign was introduced by Mitchell et al. in 1987, who considered that the inner high-signal-intensity band corresponds to granulation tissue, while the outer low-signal-intensity band corresponds to sclerotic reactive new bone [90]. However, it has been recently suggested that this “double-line sign” is related to a chemical shift artifact [91]. The center of the lesion is the necrotic area, but the signal of the fatty marrow is often preserved, especially in the early stages of the disease. AVN/bone infarction tends to be multiple, with bilateral (> 80%) or multiple joint involvements (60–90%) [92].

Fractures and subchondral collapses may occur secondary to AVN, which is clinically meaningful because this often leads to eventual joint destruction and the need for surgical intervention. Sakai et al. reported that necrotic lesions that occupy more than one-third of the condyle on coronal images are at high risk of subchondral collapse [93]. Fractures and subchondral collapse often cause severe pain, and MRI shows bone marrow edema-like signal intensity around the lesion. As the disease progresses, findings similar to those seen in SIFK, such as depression of the articular surface and subchondral fluid-filled fracture cleft, may be observed [17, 45].

### Osteochondral dissecans

**Fig. 11:**
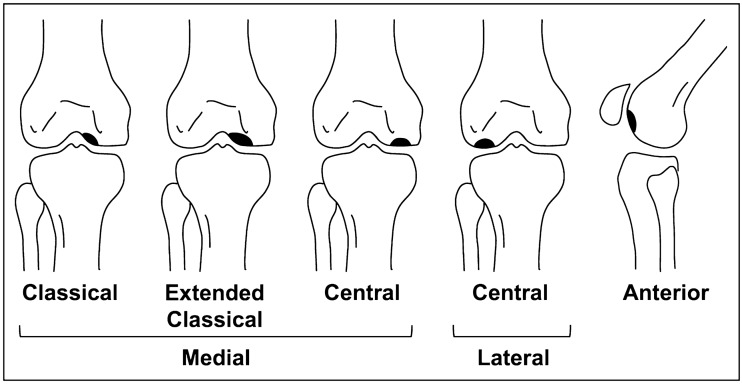
Frequent sites of OCD. The areas colored in black correspond to OCD lesions. OCD tends to occur slightly lateral to the weight bearing area of the medial femoral condyle. The classical and extended classical types both account for 75% of OCD. The lateral (13%) and medial (10%) central weight bearing surfaces of the femoral condyle are less common. OCD rarely occurs in the anterior part of the femur (2%)

OCD is a disorder that mainly affects young children and adolescents. Although the pathogenesis of this disease is still not fully understood, it is currently suggested that it results from growth disturbance of the secondary physis caused by repeated stress on the joints, including intense sports activity [17, 94, 95]. OCD sometimes develops into osteochondral fragmentation and dissection of the lesion, leading to juvenile OA of the knee [96]. In contrast to SIFK, symptoms are often absent in the early stage, and vague pain during exercise develops slowly over months or years [97]. Most knee OCD cases occur in the medial femoral condyle (85%). Compared to SIFK, OCD is more likely to occur slightly lateral to the weight bearing area of the medial femoral condyle (Fig. 11). OCD can also be found in the lateral parts of the femoral condyle (13%) and trochlea (2%) but is extremely rare in the tibia [98, 99]

**Fig. 12:**
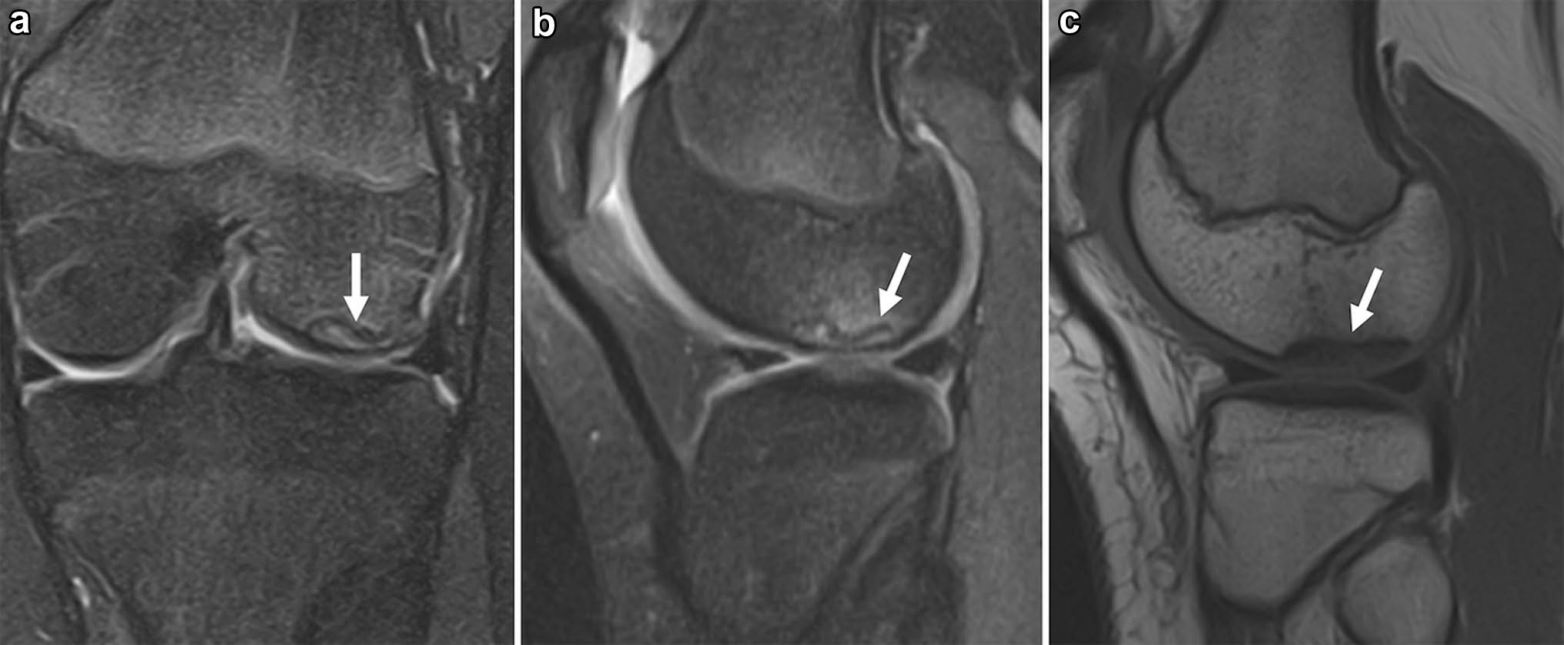
**a** Coronal FS-PDWI, **b** Sagittal FS-PDWI, and **c** T1-weighted image of a 14-year-old girl with stable OCD in the lateral femoral condyle in the left knee. The continuity of articular cartilage is preserved. There is no fluid signal or cyst formation around the lesion. Note the low signal band-like intensity observed between the lesion and the parent bone (arrows)

**Fig. 13:**
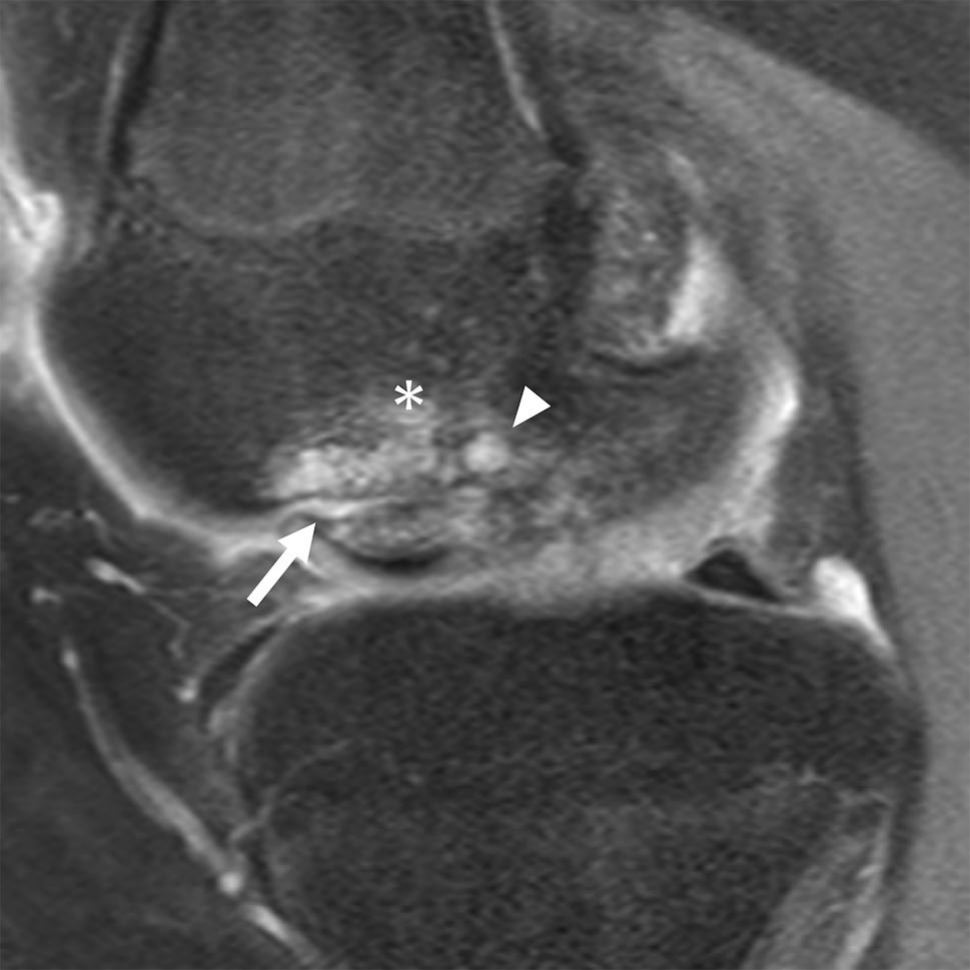
MRI of a 16-year-old boy with unstable OCD in the medial femoral condyle in the right knee. A sagittal FS-PDWI shows a rim of fluid signal intensity surrounding the OCD lesion (arrow). There is also cyst formation in the parent bone (arrowhead). Subtle bone marrow edema-like signal intensity can be observed around the cyst (asterisk)

In the early stages of OCD, radiographs are usually normal. As the disease progresses, fragmentation and detachment of the subchondral bone are observed [97, 100]. Interestingly, this process of osteochondral fragmentation begins in the deep portion under the articular surface and eventually involves the superficial articular cartilage, suggesting an “inside-out” mechanism [17]. MRI reflects this finding as abnormal signals are observed around the subchondral plate, and the continuity of articular cartilage is preserved in the early stages of the disease. A T2 low-signal band is observed between the lesion and the parent bone, which may be the initial arrest of the advancing ossification front (Fig. 12) [94]. Care should be taken because this low-signal band may resemble the fracture line of SIFK. As the disease progresses and the lesion becomes unstable, a rim of fluid signal intensity surrounding the lesion can be observed, representing the articular fluid between the lesion and the parent bone. Cyst formation beneath the lesion, detachment, or missing osteochondral fragments are also observed in advanced cases (Fig. 13) [101–103]. Bone marrow edema-like signal intensity may occasionally be observed around the lesion, but it is usually subtle compared to SIFK (Figs. 11, 12).

### Osteoarthritis

Knee OA is the most common osteochondral lesion in middle-aged and older people, and 37% of people over the age of 60 show some evidence of OA on imaging [104, 105]. It usually develops slowly. Patients complain of pain during daily activities, such as walking. As the disease progresses, it is associated with pain, even at rest and deformity of the limb [106].

**Fig. 14:**
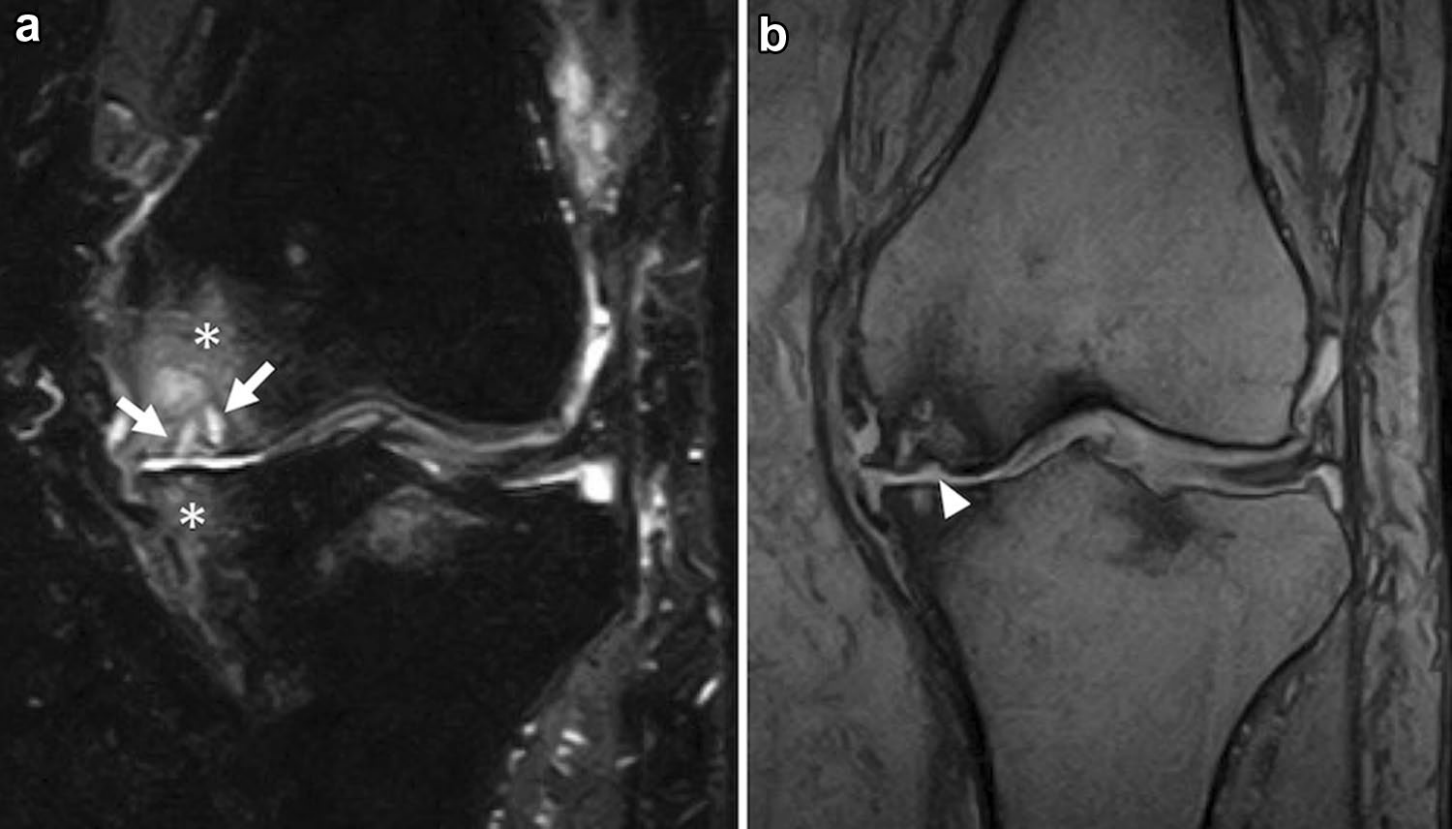
A 72-year-old female patient with knee OA. The patient complained of knee pain lasting for more than a year. **a** A coronal FS-T2WI shows severe cartilage damage and bony spur formation in the left medial femorotibial joint. Multiple subchondral cysts (arrows) and extended bone marrow edema-like signal intensity (asterisks) can be observed in the medial femoral and medial tibial condyles. **b** A coronal T2*-weighted image shows a slight deformity of the subchondral bone in the medial femoral condyle (arrowhead). This patient had been diagnosed with knee OA based on the clinical course, but the relatively old SIFK lesion was also a differential diagnosis

MRI shows thinning of the articular cartilage, sclerotic changes, formation of osteophytes, subchondral cysts, bone marrow edema-like signal intensity in the subchondral bone, joint effusion, and secondary synovitis (Fig. 14) [107, 108]. Sclerotic changes in the subchondral bone in OA may resemble the subchondral low-signal intensity area in SIFK. However, OA is often associated with other degenerative findings, such as severe cartilage damage and osteophyte formation [17]. Bone marrow edema-like signal intensity in OA corresponds to a mixture of necrosis, fibrosis, and abnormal trabeculae [43, 109] and tends to be confined to a limited area of subchondral bone, compared with SIFK. The clinical course is important for differentiating between SIFK and OA because it may be difficult to distinguish when a certain amount of time has passed since the onset of subchondral fracture.

## Conclusion

SIFK is an osteochondral lesion that commonly affects older women. Excessive contact stress related to cartilage and meniscal injury plays a major role in its development. The lesion size and the presence of osteochondral defects are associated with prognosis. The differential diagnosis of SIFK includes other osteochondral lesions, such as AVN, OCD, and OA. These diseases sometimes have similar imaging findings, and radiologists should be familiar with their imaging features and clinical presentations for appropriate management.
